# 420. Emergency Nurses’ Experiences over 1 Year of the COVID-19 Pandemic: A Qualitative Study

**DOI:** 10.1093/ofid/ofab466.620

**Published:** 2021-12-04

**Authors:** Ki Rog Lee, JaHyun Kang

**Affiliations:** 1 Asan Medical Center, Seoul, Seoul-t’ukpyolsi, Republic of Korea; 2 College of Nursing and Research Institute of Nursing Science, Seoul National University, Seoul, Seoul-t’ukpyolsi, Republic of Korea

## Abstract

**Background:**

At the frontline of fighting against the coronavirus disease 2019 (COVID-19) pandemic, emergency room (ER) nurses are faced with various challenges throughout the provision of emergency care for incoming patients without knowing their COVID-19 status. However, little is known about their work burden, exhaustion, and psychological distress in the pandemic. Therefore, to provide basic data for effective counterstrategies against future emerging infectious diseases in the ER, this study aims to understand ER nurses’ COVID-19 work experiences in depth at one tertiary hospital over 1 year.

Table 1. Summary of Qualitative Responses Regarding Emergency Room Nurses’ COVID-19 Pandemic Experience

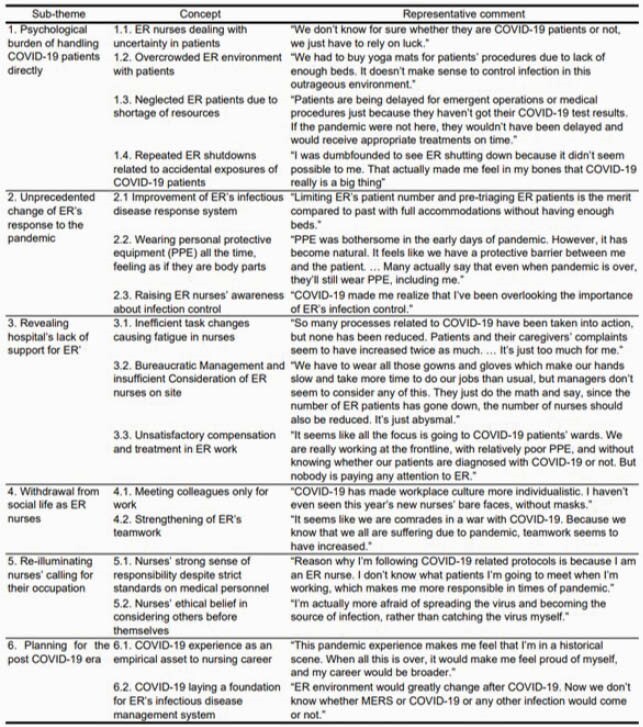

**Methods:**

This study was conducted at a 2,715-bed tertiary hospital in Seoul, Korea. Using a purposeful sampling method, we recruited 20 nurses who have worked for more than 1 year in the ER and have capacity for independent care for COVID-19 patients. With institutional review board approval, one-on-one individual, in-depth interviews were completed using a semi-structured questionnaire from February 13 to March 25, 2021. After recording and transcribing interviews, the narrative data were analyzed using a thematic analysis method.

**Results:**

The 20 participants were 29.9 years old on average with 69.2 months’ clinical experience. The overarching theme was derived as ‘COVID-19 highlighted the importance of ER’s infection control and ER nurses’ professional dedication’ covering 6 sub-themes and 16 concepts (Table 1). Sub-themes were ‘psychological burden of handling COVID-19 patients directly’, ‘unprecedented changes for ER’s response to the pandemic’, ‘revealing hospital’s lack of support for ER’, ‘withdrawal from social life as ER nurses’, ‘re-illuminating nurses’ calling for their occupation’, and ‘planning for the post COVID-19 era’.

**Conclusion:**

ER nurses experienced challenges from their drastically changed tasks, received poor compensation from the hospital, and felt pressure from social expectations towards medical personnel. However, nurses showed enough dedication towards their jobs, considered pandemic experience as a valuable asset to their future career, and maintained a positive attitude towards difficulties in ER. Providing comprehensive support for ER nurses is necessary to improve ER infection control to respond to the pandemic.

**Disclosures:**

**All Authors**: No reported disclosures

